# Delivery of Curcumin Using Zein-Gum Arabic-Tannic Acid Composite Particles: Fabrication, Characterization, and *in vitro* Release Properties

**DOI:** 10.3389/fnut.2022.842850

**Published:** 2022-03-17

**Authors:** Yiquan Zhang, Guiqiao Liu, Fazheng Ren, Ning Liu, Yi Tong, Yi Li, Anni Liu, Lida Wu, Pengjie Wang

**Affiliations:** ^1^Key Laboratory of Precision Nutrition and Food Quality, Key Laboratory of Functional Dairy, Beijing Higher Institution Engineering Research Center of Animal Product, College of Food Science and Nutritional Engineering, China Agricultural University, Beijing, China; ^2^Department of Nutrition and Health, China Agricultural University, Beijing, China; ^3^Jilin COFCO Biochemistry Co., Ltd., Changchun, China

**Keywords:** tannic acid, composite particles, stability, curcumin, bioaccessibility

## Abstract

The application of curcumin (Cur) in fat-free food is limited due to its poor water solubility, stability, and bioaccessibility. In this study, zein-gum arabic-tannic acid (zein-GA-TA) composite particles with high physical stability were fabricated to deliver Cur (ZGT-Cur). Their stability and *in vitro* release properties were also evaluated. The results showed that the thermal and photochemical stability of Cur was improved after loading into composite particles. Meanwhile, the retention rate of Cur in ZGT-Cur composite particles was enhanced compared with Z-Cur or ZG-Cur particles. Fourier transform infrared (FTIR) spectroscopy confirmed that the hydrogen bond within the particles was greatly enhanced after the addition of tannic acid (TA). The *in vitro* antioxidant activity of Cur in ZGT-Cur composite particles was higher in terms of 2,2'-azino-bis (ABTS) (93.64%) and 1,1-diphenyl-2-picrylhydrazyl (DPPH) (50.41%) compared with Z-Cur or ZG-Cur particles. The bioaccessibility of Cur in ZGT-Cur composite particles was 8.97 times higher than that of free Cur. Therefore, the particles designed in this study will broaden the application of Cur in the food industry by improving its stability and bioaccessibility.

## Introduction

Curcumin (Cur) is a yellow phenolic pigment extracted from the rhizome of turmeric (*Curcuma longa* L.) (1). Cur has been utilized as a spice, food colorant, and traditional herbal medicine in the form of turmeric, and it has been reported that Cur has significant antioxidant, anti-inflammatory, and anti-aging effects (2). However, poor water-solubility and low bioaccessibility limit its applications (3). Nanoparticles are effective carriers to improve the water solubility, stability, and bioaccessibility of Cur (4). Zein is a water-insoluble plant protein with good alcohol solubility and biocompatibility (5). Zein particles are widely used as carriers of fat-soluble nutrients.

However, zein contains a large number of hydrophobic amino acids, which promote aggregation through surface hydrophobicity. This may lead to the precipitation of zein particles and greatly limits its applications (6). To overcome this limitation, a combination of zein with a hydrophilic polysaccharide may represent a valid strategy. These hydrophilic polysaccharides include gum arabic (GA) (7), pectin (8), hyaluronic acid, (9), and carrageenan (10). It has been shown that zein-GA composite nanoparticles had higher stability over a wide pH range (7). Nevertheless, zein-GA composite particles were found to be very sensitive to a micro-environment containing salt ions, and therefore they easily aggregate and precipitate (11, 12). This was because ions will weaken the electrostatic interaction between zein and the GA and lead to the desorption of GA from the zein particles (13). Therefore, inhibiting the desorption of GA from the surface of zein particles is an effective strategy to stabilize the composite particles. As a kind of plant polyphenol, tannic acid (TA) has good water solubility. TA contains a large number of hydroxyl groups that have a strong binding ability with carbonyl groups on proteins (14). In this way, they form relatively stable hydrogen bonds, crosslinking between different protein domains (15). The introduction of hydroxyl groups might stabilize the shell-core structure of composite particles in high ionic strength environments.

In our previous work, we designed zein-gum arabic-TA (zein-GA-TA) composite particles with high physical stability based on the physicochemical properties of TA (16). The introduction of TA could link the protein part of GA crosslinked with the protein on the surface of the zein particles through hydrogen bonding. The designed zein-GA-TA composite particles could be stable to salt ions due to the introduction of TA. This study aimed to evaluate the physicochemical stability and bioaccessibility of Cur after loading into the zein-GA-TA composite particles.

## Materials and Methods

### Materials

Zein, GA, TA, Cur, porcine pepsin (≥400 U/mg), and porcine pancreatin (1,500 U/mg) were purchased from Sigma-Aldrich (St. Louis, MO, USA). Anhydrous ethanol (EtOH) was purchased from Beijing Chemical Plant (Beijing, China). The 2,2'-azino-bis (ABTS) and 1,1-diphenyl-2-picrylhydrazyl (DPPH) free radical scavenging capacity test kits were purchased from Solarbio Technology Co., Ltd. (Beijing, China), other chemicals were of analytical grade.

### The Preparation of ZGT-Cur Composite Particles

The composite particles were prepared using the method of Li et al. (7) with slight modifications. Different amount of Cur (zein:Cur = 100:1, 50:1, 20:1, 10:1, 5:1, and 2:1, w/w) were added to the 0.5% zein stock solution and stirred overnight to obtain the mixed solution of Cur and zein. The above mixture was added to 200 ml GA solution (zein:GA = 1:2, w/w). EtOH and excess water were removed by rotary evaporator at 45°C to achieve a final concentration of 0.5%, and then TA (zein:TA = 5:1, w/w) was added to the dispersion of ZG-Cur composite particles to obtain Cur-loaded zein-GA-TA composite particles. The ZGT-Cur composite particles with different mass ratios of zein and Cur are named ZGT-Cur_100:1_, ZGT-Cur_50:1_, ZGT-Cur_20:1_, ZGT-Cur_10:1_, ZGT-Cur_5:1_, and ZGT-Cur_2:1_, respectively.

### Particle Size, Polydispersity Index, and ζ-Potential

Particle size, PDI, and ζ-potential were measured by a Nano Zetasizer (Malvern, Instruments, Malvern, UK) (17). The prepared ZGT-Cur composite particle dispersion solution (100 μl) was diluted 200 times to avoid multiple scattering effects. The above indices were determined after the sample was balanced in the instrument for 120 s. All samples were measured at 25°C.

### Microstructure

The microstructure of samples was observed by a field emission scanning electron microscope (SEM) (SU8010, Hitachi, Tokyo, Japan). The freeze-dried sample was coated with gold before observation to avoid charging under the electron beam, and then images were collected with a magnification of 100,000× at 10 kV accelerating voltage (18).

### Encapsulation Efficiency (EE, %) and Loading Capacity (LC, %) of Cur

The EE and LC of Cur in ZGT-Cur composite particles were measured by spectrophotometry using a spectrophotometer (UV-8000, Shimadzu Japan) (19). ZGT-Cur samples were dissolved in 80% (v/v) aqueous EtOH and extracted by ultrasonic treatment. Then the absorbance of the solution was measured at 425 nm, and the Cur content was calculated according to the Cur absorbance-concentration standard curve. The EE and LC of Cur were calculated by Equations (1) and (2), respectively.


(1) $$\begin{array}{r} \text{EE }\left(\text{\%}\right)=\left(1-\frac{{\text{m}}_{1}}{{\text{m}}_{\text{Total}}}\right)\times 100 \end{array}$$

(2) $$\begin{array}{r} \text{LC }\left(\text{\%}\right)=\frac{{\text{m}}_{2}}{\text{M}}\times 100 \end{array}$$

where m_1_ represents the mass of free Cur, m_Total_ represents the initial total mass of Cur, m_2_ represents the mass of Cur in the nanoparticles, and M represents the mass of the sample.

### Turbiscan Stability and Storage Time Stability

Turbiscan (Formulaction, L'Union, France) was used to determine and quantify the instability mechanism of composite particle dispersion (20). The samples were measured with an interval of 10 min at 25°C for 24 h.

To evaluate the storage stability of the particles, different concentrations of ZGT-Cur composite particles were stored at 25°C for 30 days, and their particle size, PDI, and ζ-potential were measured.

### Thermal Degradation of Cur

The samples (10 ml) were put into a transparent glass bottle, heated in 25, 45, 65, and 85°C water baths for 2 h, respectively, then the above samples were cooled to room temperature (25°C), and the Cur content was calculated according to the standard curve (21). The retention rate of Cur was calculated according to Equation (3).


(3) $$\begin{array}{r} \text{Retention rate after heating }\left(\text{\%}\right)=\\ \frac{\text{ Residue mass of curcumin}}{\text{Initial mass of curcumin}}\times 100 \end{array}$$

### Photodegradation Kinetics of Cur

The samples (10 ml) were added into a transparent glass bottle and placed in a UV lightbox with a light intensity of 0.35 W/m^2^ and a constant temperature of 25°C. The residual amount of Cur in the samples was determined at 0, 30, 60, 90, and 120 min., respectively (21). The degradation kinetic parameters were fitted according to the first-order kinetic Equations (4) and (5) (22).


(4) $$\begin{array}{r} \text{Ln}\left(\text{C}/{\text{C}}_{0}\right)=\;-\text{kt} \end{array}$$

(5) $$\begin{array}{r} {\text{t}}_{1/2}=\text{ Ln}\left(2\right)/\text{k} \end{array}$$

where C represents the Cur concentration at each time point, C_0_ represents initial Cur concentration, k represents the Cur degradation rate, and t_1/2_ represents the Cur half-life.

### Fourier Transform Infrared (FTIR) Spectroscopy

The FTIR spectra of the samples were determined by an FTIR spectrometer (Nicolet iS5, Thermo Scientific, USA). Lyophilized samples (2 mg) were accurately weighed and mixed with 198 mg potassium bromide (KBr) powder. The mixture was ground into powder with a mortar, and then the powder was pressed into uniform transparent disks with a tablet press. Finally, the samples were measured with a wave-number range of 4,000–500 cm^−1^ and a resolution of 4 cm^−1^ (23).

### Fluorescence Spectroscopy

The fluorescence intensity of the samples was measured by fluorescence spectrometry (F-7000, Shimadzu, Japan) (24). The samples were diluted to a concentration of 0.2 mg/ml with deionized water before testing. The excitation wavelength was 280 nm (excitation tryptophan fluorescence), the emission wavelength range was 280–450 nm, the scan speed was 100 nm/min, the excitation and emission slit width was 5 nm, and deionized water was used as the blank.

### X-ray Diffraction

The X-ray diffraction pattern of the samples was recorded by an X-ray diffractometer (D2, AXS, Bruker, Germany). The angle range was 4–50° and the scan rate was 4°/min (25).

### *In vitro* Antioxidant Activity

The samples (0.1 g) were mixed with 4 ml of 80% (v/v) aqueous EtOH, followed by centrifugation at 1,000 rpm for 30 min, and then tested with ABTS and DPPH kits (Solarbio, Beijing, China).

### Determination of Cur Bioaccessibility

After 6 h *in vitro* gastrointestinal digestion, the digestive solution was centrifuged at 18,000 rpm for 1 h, the supernatant micelle was taken to determine the content of Cur (26). The bioaccessibility of Cur was determined according to Equation (6).


(6) $$\begin{array}{r} \text{Bioaccessibility }\left(\text{\%}\right)=\frac{{\text{M}}_{\text{micelle}}}{{\text{M}}_{\text{digest}}}\times 100 \end{array}$$

where M_micelle_ is the mass of Cur in the micelle, and M_digest_ is the mass of Cur in the digest.

### Statistical Analysis

SPSS statistics 24 software (SPSS Inc., Chicago, IL, USA) was used for one-way ANOVA, and Duncan's test was used to analyze the significance of the difference between multiple groups of samples. The significance level was *p* < 0.05. All experiments were conducted in triplicate, and the results were expressed by mean ± SD.

## Results and Discussion

### Particle Size, PDI, and ζ-Potential of ZGT-Cur Composite Particles

**Figure 1:**
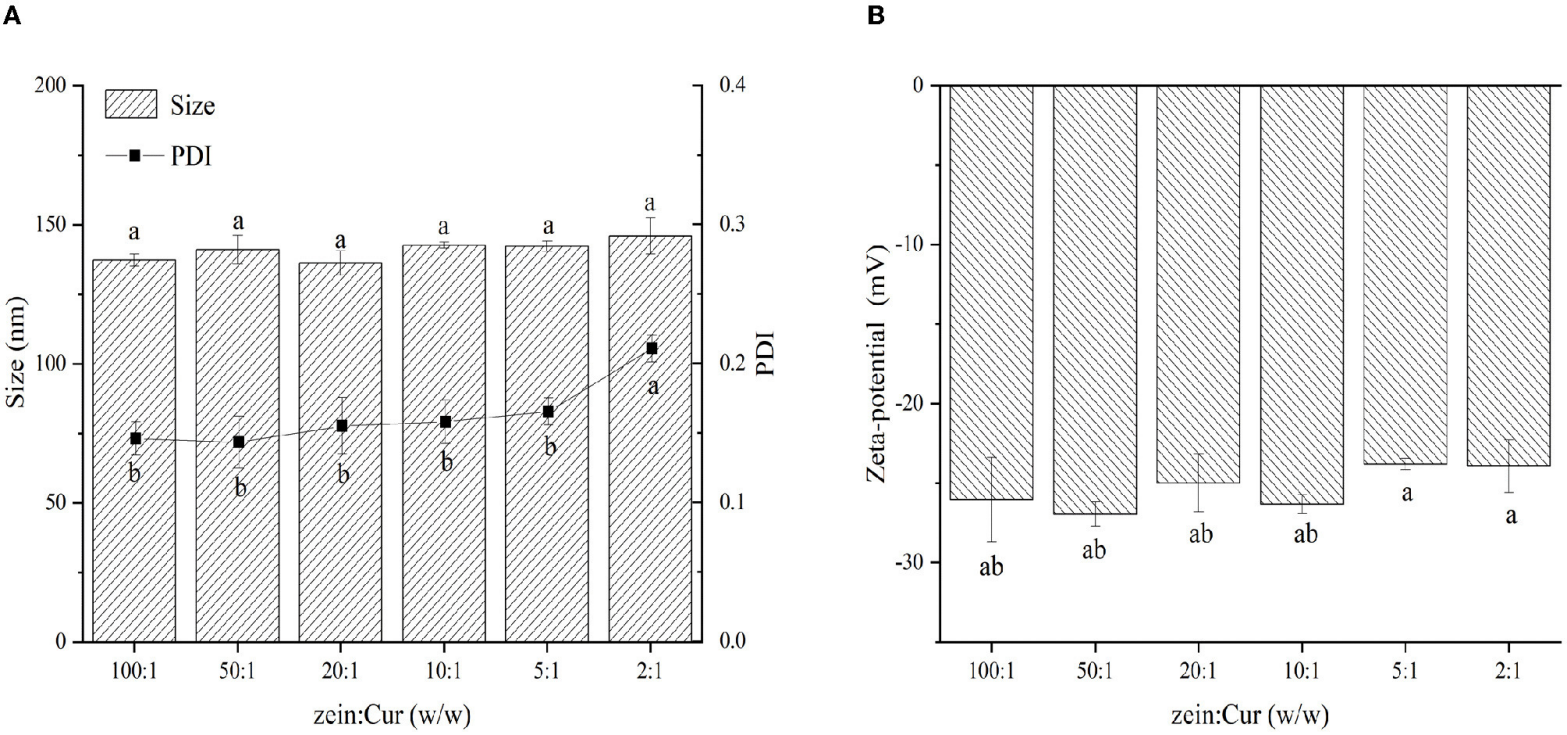
The effect of mass ratio of zein to GA **(A,B)** on the hydration diameter, polydispersity index (PDI), and zeta-potential of ZGT-Cur composite nanoparticles. Different small letters indicate a significant difference (*p* < 0.05), the same below.

The particle size, PDI, and ζ-potential of ZGT-Cur composite particles under different mass ratios of zein and Cur are shown in Figures 1A,B. When the ratio of zein:Cur was reduced from 100:1 to 2:1, the size of the ZGT-Cur composite particles was increased from 137.3 ± 2.20 nm to 145.93 ± 6.5 nm, but did not show a significant difference (*p* > 0.05). The molecular weight of Cur was small, and the addition of Cur did not affect the particle size of the composite particles (27). The trend of PDI was similar to that of the particle size, and the dispersions containing Cur were homogeneous colloidal systems. The mass ratio of zein to Cur had no significant effect on the ζ-potential of ZGT-Cur composite particles (Figure 1B), which agreed with the results reported by Chen et al. (13). The above results indicated that the ratio of zein to Cur had no impact on the particle properties of ZGT-Cur.

### Microstructure of ZGT-Cur Composite Particles

**Figure 2:**
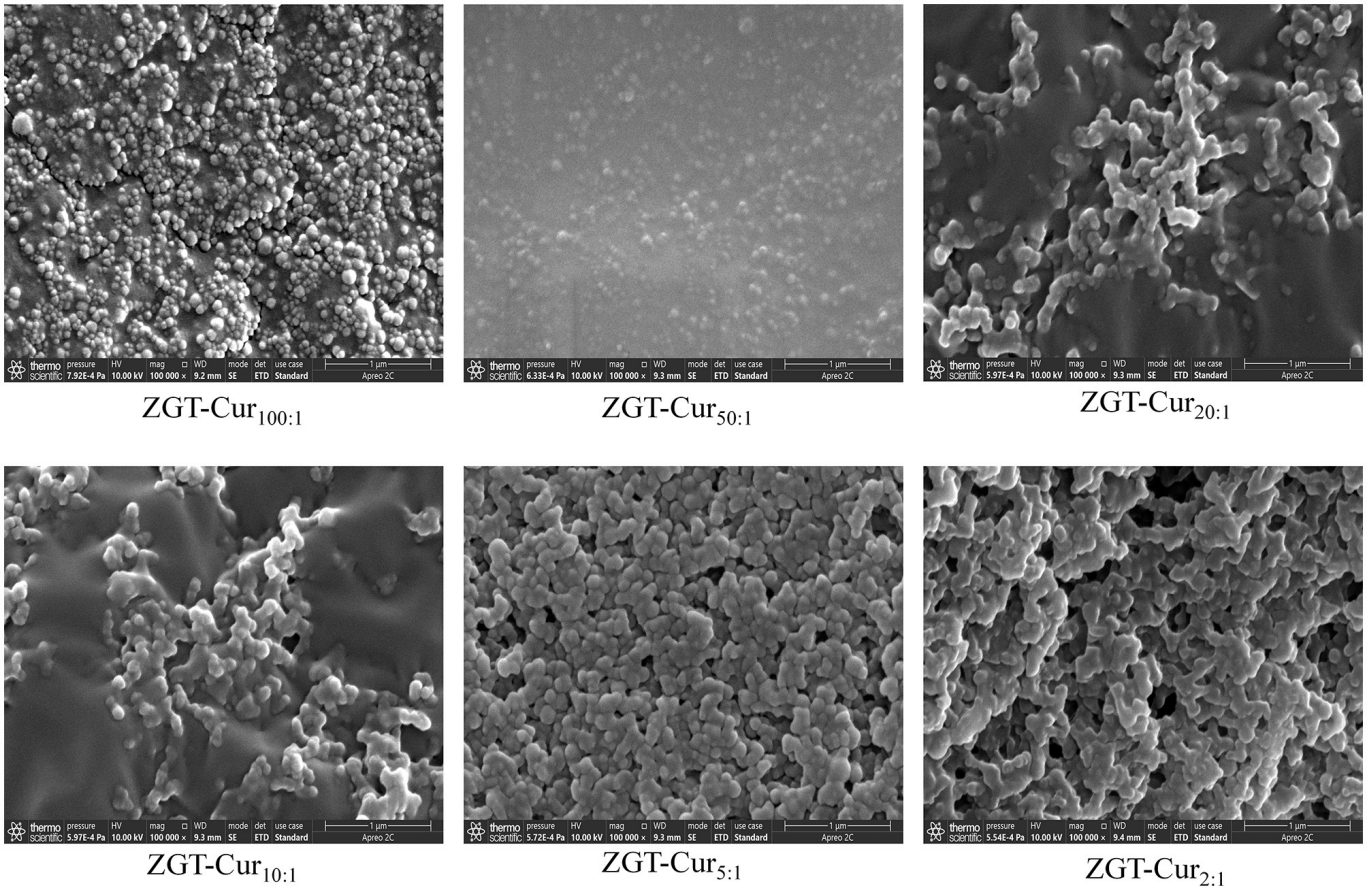
Microstructure of ZGT-curcumin (Cur) composite particles with a different mass of zein to Cur.

The effects of zein and Cur with different mass ratios on the microstructure of ZGT-Cur are observed by SEM, as shown in Figure 2. When the mass ratio of zein to Cur was reduced from 100:1 to 2:1, the particle size and micromorphology of ZGT-Cur composite particles show no statistical difference. However, the dimensions shown in Figure 2 differ somewhat from those measured using dynamic light scattering (DLS). This phenomenon may be due to the different detection principles of DLS and SEM. DLS provides the hydration diameter of particles in solution, whereas that obtained by SEM shows the images of the dried particles (28). This is consistent with that described in other literature (29). The particles readily aggregated during the drying process. The results of the microstructure also confirmed that the ratio of zein to Cur had no effect on the particle size of ZGT-Cur.

### EE (%) and LC (%) of Cur

**Figure 3:**
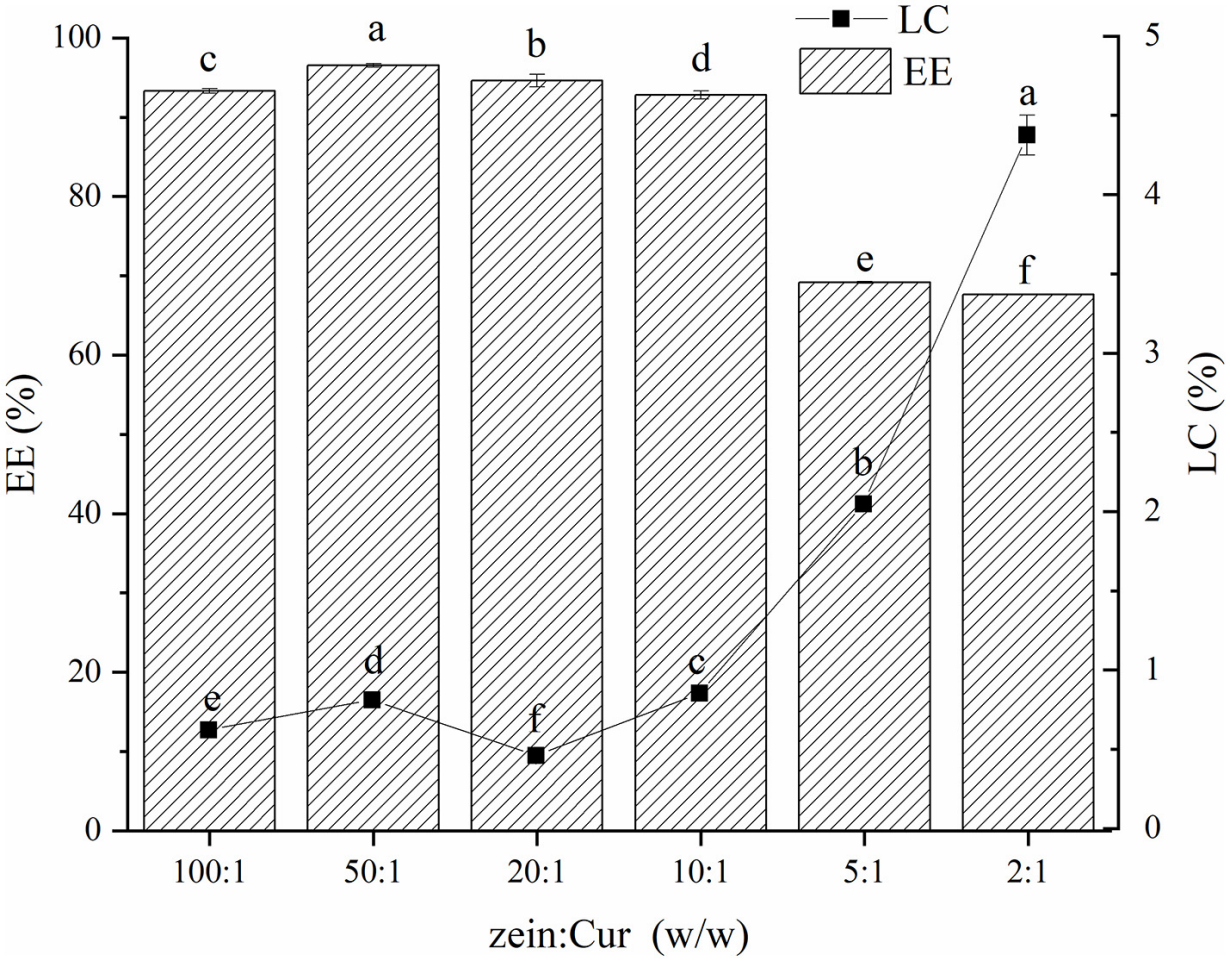
Encapsulation efficiency and loading capacity of curcumin (Cur) in ZGT-Cur composite nanoparticles at different mass ratios of zein to curcumin.

The EE and LC are often used to evaluate the application potential of the carrier delivery system. Figure 3 shows the EE and LC of Cur in the ZGT-Cur composite particles. The EE of the composite particles showed a decreasing trend as the mass ratio of zein to Cur decreased (Figure 3). The excessive addition of Cur exceeded the encapsulation ability of the composite particles, resulting in a decrease in EE (10). When the mass ratio of zein to Cur was 100:1, the EE and LC were 93.32 ± 0.29% and 0.62 ± 0.01%, respectively. However, when the mass ratio of zein to Cur was 2:1, the EE was decreased to 67.63 ± 0.06%, and the LC was increased to 4.38 ± 0.13%. As the addition of Cur was increased, the LC of the composite particles was also increased, which is consistent with the results of Chen et al. (30).

### Storage Stability of Composite Particles

**Figure 4:**
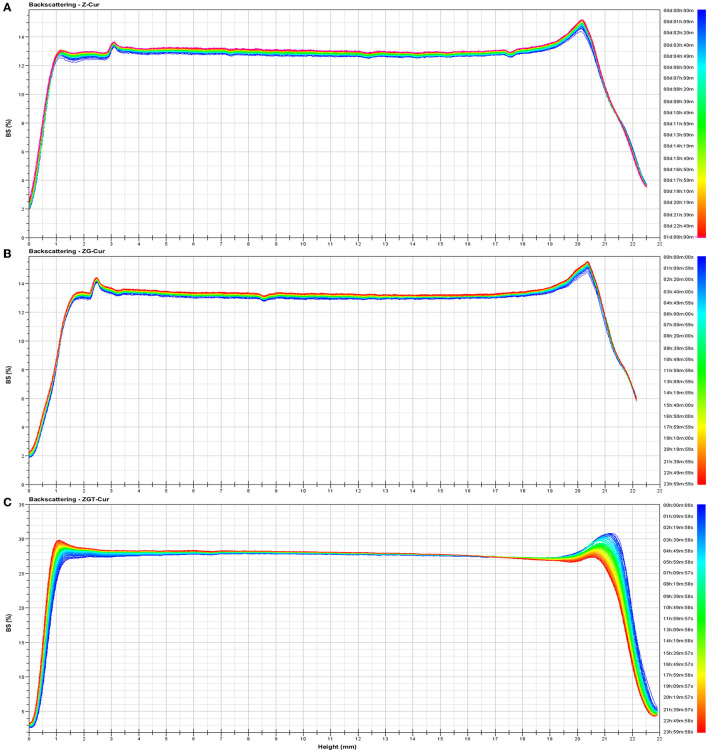
Backscattering changes of Z- curcumin (Cur) **(A)**, ZG-Cur **(B)**, and ZGT-Cur **(C)** composite particle dispersions. Moreover, x-axis means height (mm), y-axis means BS (%).

Figure 4 shows the backscattered light intensity of ZGT-Cur, Z-Cur, and ZG-Cur. As can be seen from Figure 4, the slight increase of backscattered light intensity of Z-Cur and ZG-Cur particles at the bottom and on the top of the bottle indicated that a small number of particles have gathered. The backscattered light intensity of ZGT-Cur composite particles did not change significantly, indicating that the particles did not precipitate or aggregate.

**Figure 5:**
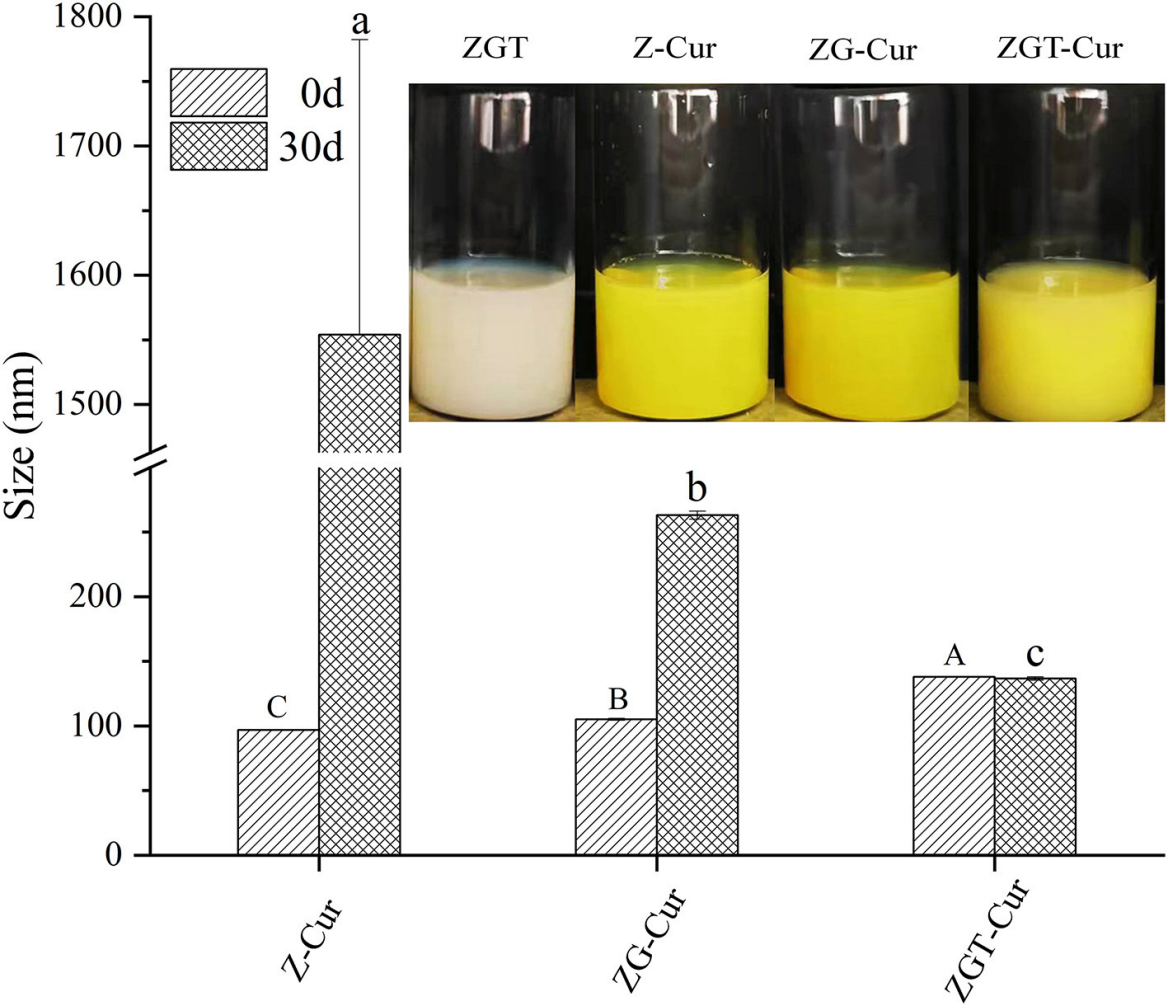
Effect of different storage times (0 and 30 days) on composite particle size.

Figure 5 shows the particle size of ZGT-Cur, Z-Cur, and ZG-Cur during storage for 30 days. The particle sizes of ZGT-Cur, Z-Cur, and ZG-Cur composite particles were significantly different (*p* < 0.05; Figure 5). The size of Z-Cur particles was increased from 96.94 ± 0.26 nm at 0 day to 1,544.00 ± 228.11 nm at 30 days (*p* < 0.05) and precipitation was observed at the same time. The size of the ZG-Cur particles was increased from 105.13 ± 0.68 nm to 263.10 ± 3.12 nm (*p* < 0.05). The stability of the nanoparticles was destroyed to some extent during storage, and the increase in particle size during storage was due to particle aggregation and expansion (31). However, the size of the ZGT-Cur composite particles did not change significantly during storage, and the particle size of ZGT-Cur was 123.97 ± 1.27 nm at 30 days. The above results showed that the stability of ZGT-Cur was better than Z-Cur and ZG-Cur, and TA had a certain ability to resist aggregation.

### Thermal Degradation of Cur

**Figure 6:**
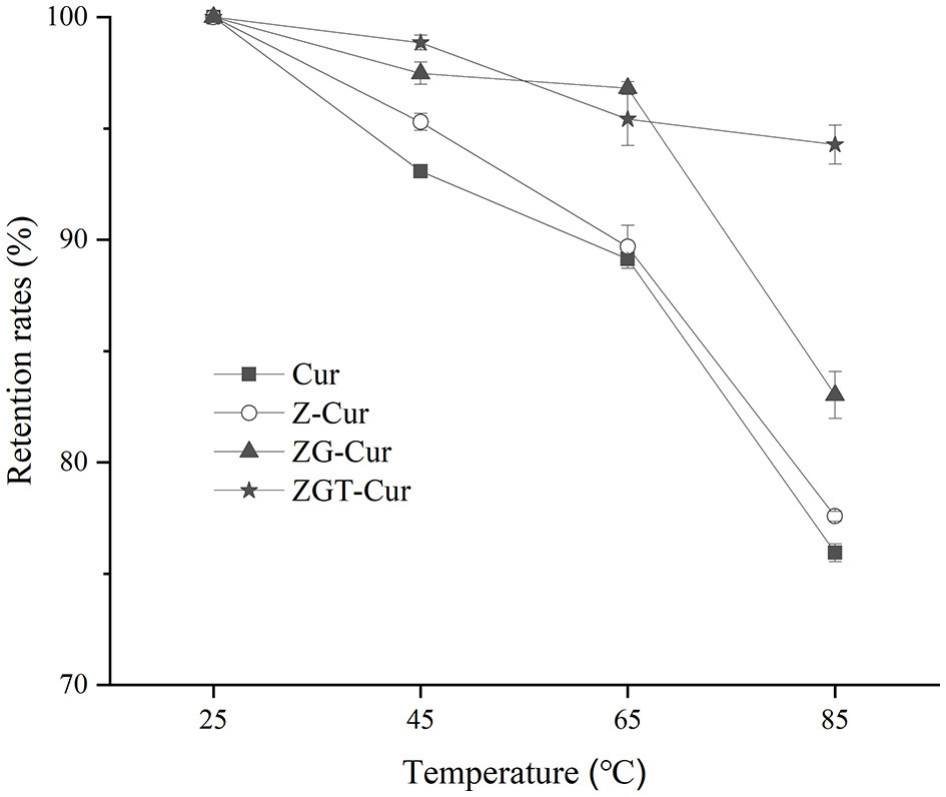
Effect of different temperatures (25, 45, 65, and 85°C) on retention rates of Cur in composite particles.

Thermal treatment is an important procedure in food processing. Heat treatment has an important effect on the content of functional components (32). As can be seen from Figure 6, the retention rate of Cur in all samples has decreased gradually as the heating temperature is increased. The retention rates of free Cur at 45, 65, and 85°C were 93.1, 89.1, and 73.9%, respectively. When GA was included in the particles, the retention rates of Cur in ZG-Cur were improved from 77.6 to 83.0% at 85°C. It has also been reported that the addition of polysaccharides can improve the retention rate of Cur (21). When TA was added, the retention rates of Cur in ZGT-Cur composite particles were further improved to 98.8, 97.67, and 94.3% at 45, 65, and 85°C, respectively. The above results showed that TA could improve the thermal stability of Cur in ZGT-Cur composite particles. Similar results can also be observed in a previous study (33).

### Photodegradation

**Figure 7:**
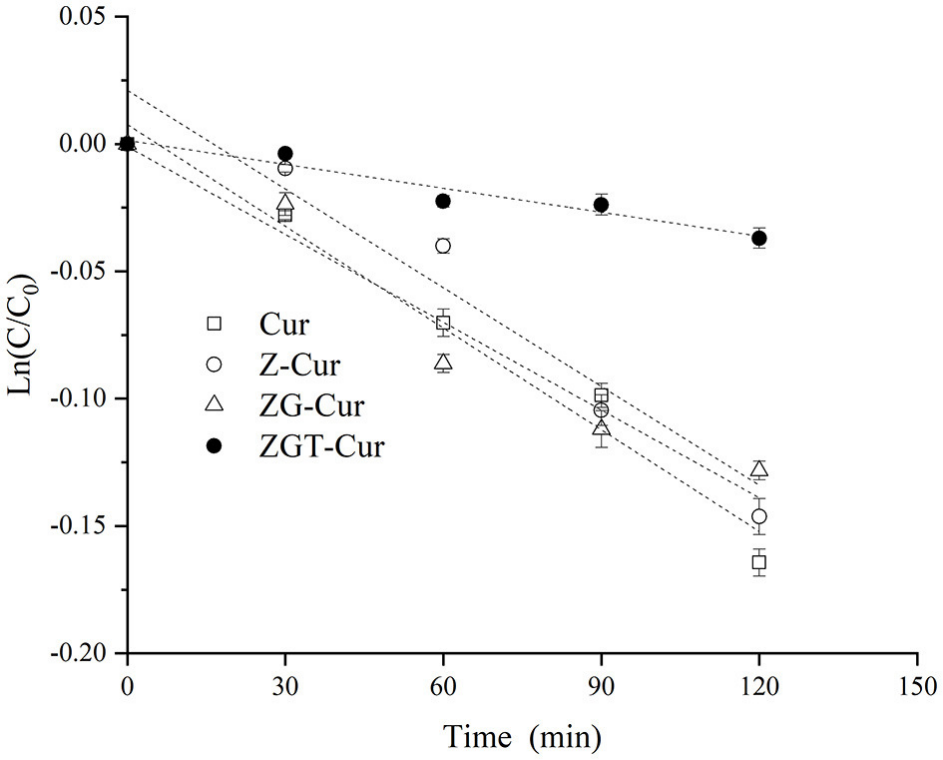
First-order kinetic curve fitting of curcumin degradation during UV irradiation.

As shown in Figure 7, the first-order kinetic curves of each sample are different. The fitted curves of Cur, Z-Cur, and ZG-Cur composite particles had larger slopes, while the fitted curve of ZGT-Cur composite particles had a smaller slope and the curve was less steep. This indicated that the degradation rate of Cur in the ZGT-Cur composite particles containing TA was slower.

The coefficient of determination (R^2^) of the fitting curve of each sample was >0.95, and the degradation rate of Cur for each sample was between 0.00030 and 0.00118 min^−1^ (Table 1). The degradation rate of free Cur was 0.00118 min^−1^, and the half-life was 586 min. When Cur was encapsulated by zein, the half-life of Cur in Z-Cur increased significantly to 621 min (*p* < 0.05). The aromatic groups and disulfide bonds in zein molecules can absorb UV light, thus enhancing the UV-light resistant ability of Cur (34). When zein was further encapsulated with GA, the degradation rate of Cur in ZG-Cur was slowed down and the half-life was increased significantly to 680 min (*p* < 0.05). This was because the addition of GA provided more physical barriers for Cur. Similar findings were also reported by Yu et al. (35) who pointed out that Cur encapsulated in composite particles showed better photostability. The half-life of Cur in ZGT-Cur composite particles was 2,312 min, which was 2.9 times that of ZG-Cur composite particles. These results indicated that the degradation rate and half-life of Cur can be significantly slowed down by encapsulation and the UV stability of Cur can be further improved when TA is included in the composite particles. Similar results were reported for the UV-light resistant ability of Cur, which was also improved after embedding (21).

**Table 1:** Parameters of the first-order model for curcumin degradation during UV irradiation.

**Sample**	**R^**2**^**	**Degradation rate (min^**−1**^)**	**Half-life period (min)**
Cur	0.974	0.00118 ± 0.000015^a^	586 ± 8^d^
Z-Cur	0.957	0.00112 ± 0.000015^b^	621 ± 9^c^
ZG-Cur	0.984	0.00102 ± 0.000010^c^	680 ± 7^b^
ZGT-Cur	0.976	0.00030 ± 0.000010^d^	2312 ± 77^a^

*The different small letters indicate a significant difference (p < 0.05)*.

### FTIR Spectroscopy Analysis

**Figure 8:**
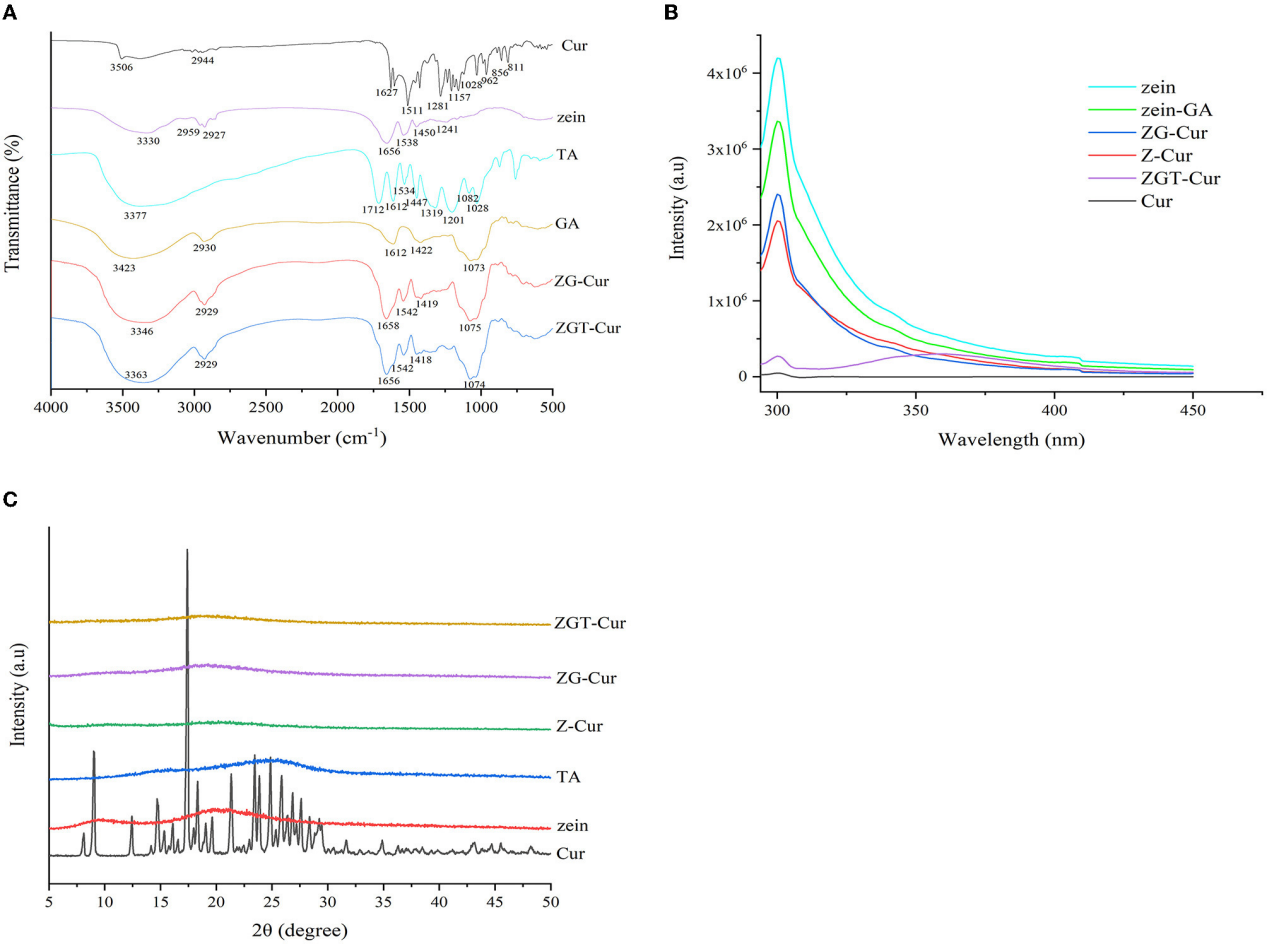
Fourier transform infrared **(A)**, fluorescence spectrums **(B)**, and X-ray diffraction **(C)** spectra of the materials used to prepare the ZGT-curcumin (Cur) particles.

The interactions of Cur, zein, GA, and TA in the preparation of ZGT-Cur composite particles can be evaluated by the peak shift and peak intensity of the FTIR spectra. As shown in Figure 8A, Cur has characteristic peaks at 3,506, 1,627, 1,428, 1,281, and 1,028 cm^−1^. This was consistent with the results reported by Feng et al. (23). There were no Cur characteristic peaks in the peak spectra of ZG-Cur and ZGT-Cur composite particles, indicating that Cur was encapsulated by the biopolymers. TA had a characteristic peak at 3,377 cm^−1^ and ZG-Cur composite particles had a wide peak at 3,346 cm^−1^. However, ZGT-Cur composite particles containing TA had an absorption peak at 3,362 cm^−1^, indicating that TA and ZG-Cur were bound by hydrogen bonding (36). The introduction of TA did not change the N-H and C-N tensile vibration of zein and ZG-Cur secondary amide groups at 1,542 cm^−1^, indicating that TA did not affect the secondary structure of zein. ZGT-Cur contained the characteristic peak of GA at 1,074 cm^−1^, but did not contain the characteristic peak of TA at the wavelength of 1,534–1,712 cm^−1^. Therefore, it was speculated that TA was wrapped inside by GA and interacted with the zein.

### Fluorescence Property

As shown in Figure 8B, zein has a strong fluorescence intensity at 300 nm. Compared with zein, the fluorescence intensity of zein-GA composite particles was decreased, indicating that the combination of GA to zein was reduced the fluorescence intensity of zein. When loading Cur by zein or zein-GA, a decrease of zein fluorescence intensity was also observed, which also showed that there was a combination of zein, GA, and Cur. The fluorescence quenching of the tyrosine of zein was caused by the specific interaction between the fluorophore and Cur (24). However, compared with the effect of GA on zein fluorescence intensity, Cur had a greater effect on the zein fluorescence intensity. The result indicated that the interaction force between zein and Cur was stronger. After TA was added to ZG-Cur, the fluorescence intensity of ZGT-Cur composite particles was further reduced at 300 nm and a new emission peak was generated at 360 nm, which again showed that TA bound to zein. Hydrogen bonds were formed mainly between the phenolic hydroxyl group of TA and the amide group of the protein (37).

### X-ray Diffraction

The physical state of Cur before and after embedding can be detected by X-ray diffraction. As shown in Figure 8C, there are two wide diffraction peaks appearing at 2θ = 9.5° and 2θ = 19.7° in the XRD profile of zein, and TA had a wide diffraction peak at 25.2° indicating that zein and TA are amorphous structures. However, the characteristic diffraction peaks of Cur did not appear in the X-ray diffraction spectra of ZGT-Cur, ZG-Cur, and Z-Cur composite particles, and the peak intensity produced by the composite particles was very weak. The above results showed that the structure of Cur changed from crystalline to amorphous, indicating that the interaction between Cur and the composite particles had an impact on the crystal form. Similar findings were also found in the study of zein-carrageenan particles embedded with Cur (38).

### *In vitro* Antioxidant Activity

**Figure 9:**
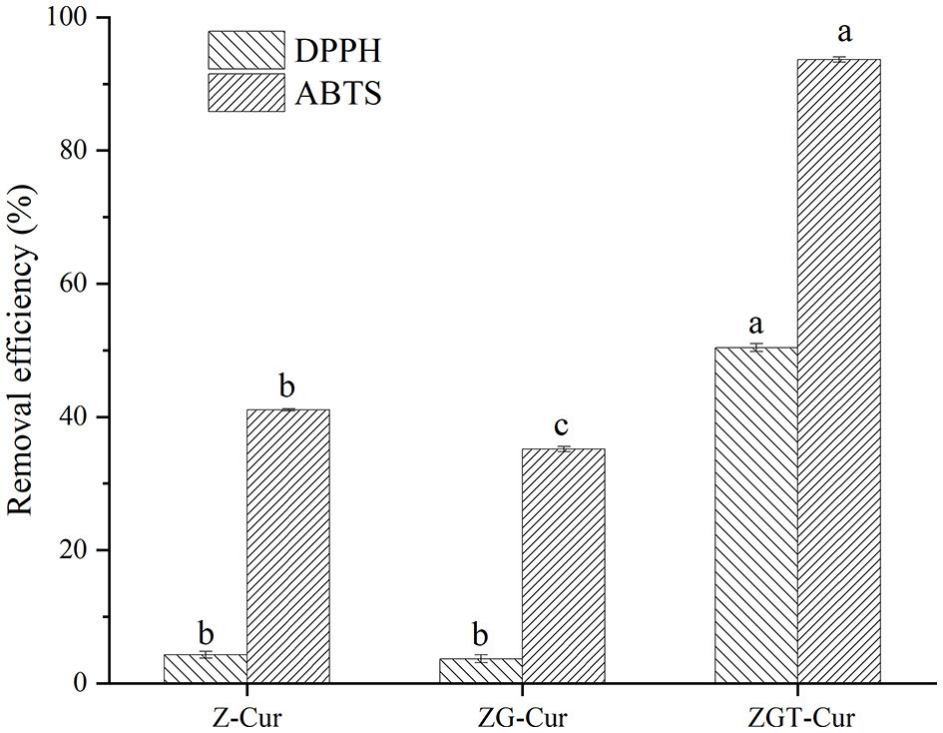
1,1-diphenyl-2-picrylhydrazyl (DPPH) and 2,2'-azino-bis (ABTS) removal efficiency of composite particles.

In this study, the free radical scavenging abilities of six different composite particles were evaluated. The DPPH radical scavenging rates of Z-Cur and ZG-Cur were 4.26 and 3.29%, respectively (Figure 9). The DPPH free radical scavenging rate of ZGT-Cur composite particles was hugely raised to 50.41% (*p* < 0.05). The trend of the ABTS radical scavenging ability of the composite particles was similar to that of the DPPH radical scavenging ability. It had been shown that encapsulation of Cur with nanoparticles was an effective way to improve its antioxidant capacity, because the composite particles promoted the structure of Cur conjugated diene and provided protons for free radicals (39). The content of Cur within the composite particles was similar, therefore, the addition of TA can improve the antioxidant capacity of Cur-loaded composite particles. Meanwhile, TA and Cur might have an antioxidant synergistic effect. As previously reported, TA-crosslinked nanoparticles enhanced the antioxidant capacity of eugenol (33).

### *In vitro* Bioaccessibility

**Figure 10:**
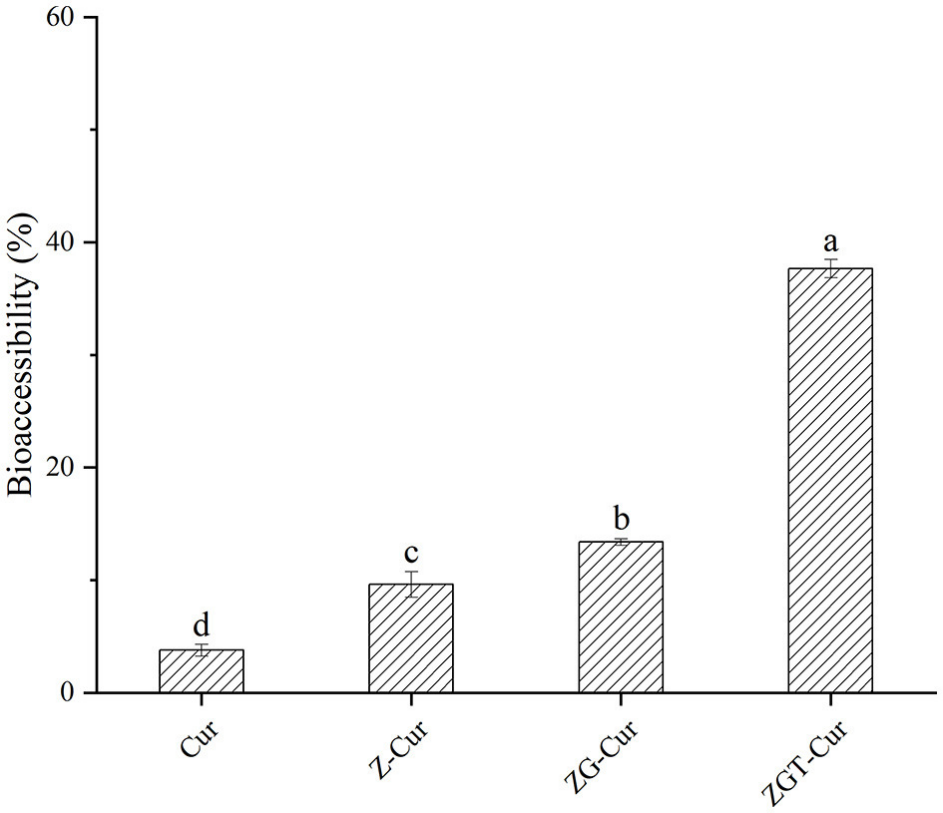
Bioaccessibility of curcumin (Cur) in composite particles.

Cur is highly hydrophobic so that it can only be absorbed after dissolution in gastrointestinal fluid. The gastroenteric fluid contains surfactants, bile salts, and enzymes that together form mixed micelles during the dissolution of Cur (40). As can be seen from Figure 10, after 6 h of simulated *in vitro* digestion, the bioaccessibility of free Cur is 3.78%. When Cur was embedded with zein, the bioaccessibility of Cur was increased to 9.62%. After embedding Cur with zein-GA composite particles, the bioaccessibility of Cur was further improved to 13.38%, which was 2.54 times higher than that of free Cur. When TA was added, the bioaccessibility of Cur in ZGT-Cur composite particles was increased to 37.67%. It could be that the hydrogen bond interaction between TA, zein, GA, and Cur can make more Cur exist in ZGT-Cur composite particles, and the stable carrier structure can play a key role in controlled release. It was found that the combination of TA and pectin may enhance the absorption of Cur by inhibiting the function of osmotic glycoprotein (41).

## Conclusion

In this article, Cur-loaded zein-GA-TA composite particles were prepared, and the particle characteristics, bioaccessibility, and antioxidant properties of ZGT-Cur composite particles were studied. As expected, the ZGT-Cur composite particles significantly improved the physical stability, antioxidant activity, thermal, and photochemical stability of Cur, compared to Cur-loaded in zein or zein-GA particles. Overall, ZGT-Cur composite particles delivery systems provide insight into improving the bioaccessibility of Cur, which is worthy of further exploration.

## Data Availability Statement

The original contributions presented in the study are included in the article/supplementary material, further inquiries can be directed to the corresponding authors.

## Author Contributions

YZ and GL conceptualized the manuscript. FR contributed to methodology. NL contributed to investigation. PW contributed to data curation and project administration. YT contributed to formal analysis. YZ wrote the original draft. YL wrote the review and edited the manuscript. AL contributed to resources and reviewed. LW supervised the work. All authors contributed to the article and approved the submitted version.

## Funding

This research was funded by the National Natural Science Foundation of China (no. 3190625).

## Conflict of Interest

YT, YL, AL, and LW are employed by Jilin COFCO Biochemistry Co., Ltd. The remaining authors declare that the research was conducted in the absence of any commercial or financial relationships that could be construed as a potential conflict of interest.

## Publisher's Note

All claims expressed in this article are solely those of the authors and do not necessarily represent those of their affiliated organizations, or those of the publisher, the editors and the reviewers. Any product that may be evaluated in this article, or claim that may be made by its manufacturer, is not guaranteed or endorsed by the publisher.
